# Dietary intervention for children and adolescents with familial hypercholesterolaemia

**DOI:** 10.1186/s13052-023-01479-8

**Published:** 2023-06-22

**Authors:** Maria Elena Capra, Giacomo Biasucci, Elisa Crivellaro, Giuseppe Banderali, Cristina Pederiva

**Affiliations:** 1 Centre for Paediatric DyslipidaemiasPaediatrics and Neonatology Unit, Guglielmo da Saliceto Hospital, University of Parma, 29121 Piacenza, Italy; 2 Department of Translational Medical and Surgical Sciences, University of Parma, 43126 Parma, Italy; 3 Società Italiana Di Nutrizione Pediatrica (SINUPE), 20126 Milan, Italy; 4 University of Milan, 20122 Milan, Italy; 5 Clinical Service for DyslipidaemiasStudy and Prevention of Atherosclerosis in ChildhoodPediatrics Unit, ASST-Santi Paolo E Carlo, 20142 Milan, Italy

**Keywords:** Familial hypercholesterolaemia, Children, Dietary pattern, Healthy lifestyle, Mediterranean Diet

## Abstract

Familial hypercholesterolaemia (FH) is a frequent genetic disorder characterised by high plasma levels of total and LDL-cholesterol and premature atherosclerosis. If left untreated, affected subjects have a high risk of cardiovascular disease, as they are exposed to very high levels of LDL-cholesterol from birth. Healthy dietary habits and lifestyle are the first treatment option and, if started from childhood, represent a milestone in the prevention of atherosclerotic disease, both as a starting point and in combination with drug therapy. In this work, based on the main consensus documents available so far, we have evaluated the most up-to-date indications of the dietetic-nutritional intervention for the treatment of FH, delving into the peculiar aspects of the diet of the child/adolescent affected by FH. After an analysis of the macro- and micronutrients and the most common dietary patterns currently recommended, we highlighted some practical aspects, some frequent errors and some risks we could fall into when dealing with paediatric nutritional treatment. In conclusion, the dietary intervention for the child/adolescent with FH is a complex task, that should be individualised and tailored taking into account, first of all, the nutritional adequacy for growth and development, but also the multiple aspects linked to the child/adolescent's age, tastes and preferences, the family they belong to, the socio-economic context and the Country they live in.

## Main Text

### Introduction

#### Familial Hypercholesterolaemia and atherosclerosis

Familial hypercholesterolaemia (FH) is a primitive dyslipidaemia characterised by genetic mutations involving cholesterol metabolism, causing an increase in total cholesterol (TC) and low density lipoprotein cholesterol (LDL-C) plasma levels and, therefore, an increased risk of atherosclerosis [1]. In its heterozygous form, FH involves 1 in 200–250 subjects in the general population [2]. FH is already present in childhood, but most of the time it is asymptomatic until adult age. FH is a common genetic disease causing hypercholesterolaemia, that is one of the main cardiovascular disease (CVD) risk factors, and it highly increases morbidity and mortality rates in undetected and untreated patients. Early detection and treatment of subjects with FH, starting from the first years of life, helps “gaining decades of life” [3]. Unfortunately, lipid and CVD prevention issues are yet poorly known and debated in the young adult population [4]. Knowledge of the problem, prompt and adequate detection and treatment of subjects with FH in childhood, identification of other CVD risk factors, such as overweight or obesity, hypertension, diabetes and high lipoprotein (a) levels [5] are milestones in the management of paediatric subjects with FH [3, 6, 7]. Nutritional and lifestyle intervention are first line treatments that have to be started as soon as possible, managed by expert Paediatric Lipidologists at a dedicated Lipid Clinic, involving all the family. Heart-safe nutritional and lifestyle habits will accompany the child throughout adolescence to adulthood. In most cases, pharmacologic therapy is necessary for FH children, starting from age six to ten, according to different national guidelines and to clinical and biochemical presentation of hypercholesterolaemia [1, 8, 9].

#### Evidence on nutritional intervention in patients with FH

Nutritional intervention is a milestone in the treatment of subjects with FH, together with pharmacologic treatment when needed, according to current guidelines [1, 6]. However, adults’ recommendations cannot be applied *tout court* in childhood, as nutritional intervention in paediatric patients should not only ensure an improvement of lipid profile, but also their adequate growth and neuro-development [10]. Prudent low-fat diet in childhood is effective and safe in terms of growth, as shown in the DISC Study and in the STRIP Study [11, 12]. Prudent low-fat diet in paediatric patients with FH can lower TC and LDL-C plasma levels to 10–15% of the starting values [13]. In this context, pharmacologic lipid lowering therapy can be delayed or started at a lower dose.

The aim of our study is to analyse the main consensus statements and available documents on management and treatment of patients with FH, focusing on nutritional treatment of children and adolescents. The MEDLINE–PubMed database was searched from 1992 to 2023 to collect the literature. The following combinations of keywords were used: “familial hypercholesterolemia” AND “nutrition” OR “ dietary” AND “children” OR “pediatric” OR “pediatric” OR “adolescent”. We selected European and USA Documents.

The search was limited to English-language journals, Italian National Documents and full papers only.

### Lifestyle and nutrition to improve lipid profile: what do we know?

The role of nutrition in CVD prevention is a well-known and consolidated issue in literature [14–16]. Nutritional and dietetic interventions are positive epigenetic factors that can modulate and modify some important CVD risk factors, such as dyslipidaemia, high glycaemic plasma levels and hypertension [17–20].

In the past few years, research in nutritional field has been focused mainly on dietary patterns’ effect on CVD risk factors [21, 22] rather than on the effect of single nutritional component on plasma lipids [23]. A few studies have reported that a nutritional intervention, based on a high weekly intake of fruit, vegetables, pulses, whole food, yogurt, fish, olive oil, and on a low weekly intake of red meat, processed meat, sugar, salt, is associated with a reduction of CVD [24]. What is more, animal derived lipids substitution with vegetal derived lipids [25] and with long chain polyunsaturated fatty acids (LCPUFA) has been linked to reduction of cardiovascular diseases [26, 27]. In the EAS/ESC guidelines [8], the Mediterranean Diet is recommended as a dietary model as it has been associated, in adult subjects, with a reduced CVD incidence in epidemiological studies [28–30].

We will briefly analyse macronutrients and lifestyle influence on lipid profile. We will also analyse the main nutritional models proposed for the treatment of dyslipidaemia.

#### Lipids

Saturated fatty acids (SFAs) are the dietetic components that mostly influence plasma low density lipoprotein cholesterol (LDL-C) levels, with an average increase of 0.8 to 1,6 mg/dl of plasma LDL-C levels for every percentage point of energy derived from SFAs [19, 31]. According to international guidelines, saturated fat intake should not exceed 10% of total daily caloric intake for the general population and it should be lower than 7% of total daily caloric intake for subjects with hypercholesterolaemia [8, 32, 33]. In a similar way, trans fatty acids (TFAs) determine an increase in LDL-C but, differently from SFAs, they also cause a reduction in HDL-C [34]. Trans- fatty acids are contained in meat, in dairy products and especially in elaborated bakery products, where they are represented by partially hydrogenated fatty acids in vegetal oils, accounting for 80% of hydrogenated lipid amount [35]. Trans- fatty acids use in nutritional field is regulated internationally and, according to WHO, it must not exceed 2% of total fats in foods [36–38].

On the contrary, LCPUFAs have a positive effect on lipid profile, causing a reduction in LDL-C and an increase in HDL-C plasma levels. LCPUFAs are mainly contained in fish (especially blue fish), seeds, pulses, soy and nuts. LCPUFAs should be used instead of saturated fats contained in butter and lard [39, 40], although the optimal intake of (n-3) and (n-6) LCPUFAs is not yet detailed [41, 42].

Finally, in subjects with hypercholesterolaemia, the daily dietary cholesterol intake should not exceed 300 mg per day [8].

#### Carbohydrates and fibres

Dietary carbohydrates mainly influence plasma TG and HDL-C levels, whereas they have a neutral effect on TC and LDL-C levels [19]. Soluble fibres, contained in pulses, fruit, vegetables and whole cereals (oat and barley) have a cholesterol-lowering effect and they represent a good energy source that can balance the effect of lipid reduction [43, 44]. According to guidelines [8], carbohydrates daily intake should be between 45 and 55% of total caloric daily intake. For subjects following a prudent low lipid diet, daily fibres amount of 25 to 40 g with a percentage of 7 to 13% of soluble fibres is generally well tolerated and can help reaching cholesterol goal. A recent meta-analysis of epidemiological studies has highlighted the presence of a U-shaped relationship between carbohydrates intake and mortality, showing an increase in mortality rate when carbohydrates daily intake is lower than 40% or higher than 70% of total daily caloric intake [45].

Added sugars (in addition to simple carbohydrates naturally present in fruits and dairy products) should not exceed 10% of total daily caloric intake and should be further reduced in subjects with overweight or obesity, high TG levels, metabolic syndrome and/ or diabetes mellitus [46]. A high simple carbohydrates dietetic intake can cause an increase in TG plasma levels, as well as an elevated fructose intake (higher than 10% of total daily caloric intake), with a dose-dependent effect (e.g.: fructose intake accounting for 15–20% of total daily caloric intake can cause a 30 to 40% increase of TG plasma levels) [47, 48].

#### Body weight reduction

Body weight (BW) reduction can positively influence TC and LDL-C plasma levels, but with a limited effect (in obese subject a weight loss of 10 kg has been linked to a reduction of 8 mg/dl in LDL-C levels), whereas BW reduction has a greater effect on TG plasma levels, on insulin resistance and on HDL-C levels (e.g.: for every kg of BW lost, an increase of 0.4 mg/dl of HDL-C plasma levels has been reported) [49, 50].

#### Physical activity

Daily physical activity has been linked to a reduction in TG and increase in HDL-C plasma levels: 25–30 min of daily walk can make HDL-C plasma levels raise from 3.1 to 6 mg/dl. Physical activity has a slight effect on LDL-C reduction [51, 52].

**Fig. 1:**
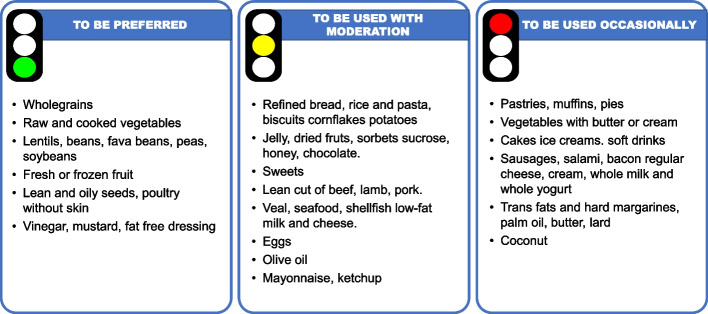
Frequency of different food consumption according to their effect on CVD risk, adapted from EAS/ESC guidelines 2019 [8]

**Fig. 2:**
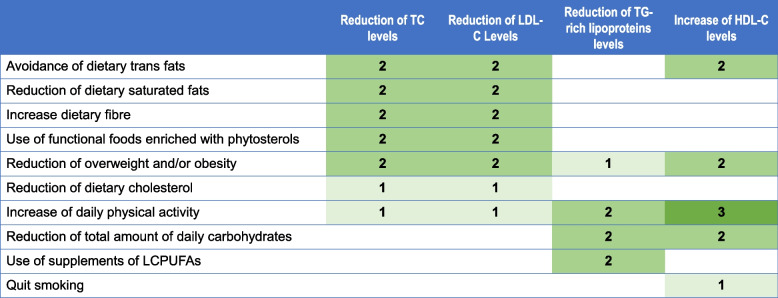
Effect of nutritional and lifestyle habits on lipid profile. 3 stands for a > 10% variation in specific lipid fraction, 2 for a 5–10% and 1 for a < 5% variation. Empty box means that there is no effect on lipid profile, adapted from EAS/ESC guidelines 2019 [8]

In Fig. 1 and 2 (adapted from EAS/ESC guidelines [8]) we have summarised the effect of dietary and lifestyle habits on lipid profile.

### Dietetic models

In the past few years, dietetic models have been widely analysed, focusing mainly on dietary patterns instead of each single nutrient influence on lipid profile. The healthy-heart dietary patterns for the treatment of subjects with hypercholesterolaemia have been modulated according to local dietary habits and cultural heritage of each Country, so as to make them more accepted and easier to follow in the real life. Mediterranean Diet is the main nutritional model referred to in International Guidelines as being effective in terms of CVD prevention [8]. The Traditional Mediterranean diet is defined as the nutritional model mainly adopted in the olive tree-growing areas of the Mediterranean region back to the early 1960s. It is characterised by an elevated consume of vegetables, fruits, pulses, and cereals (mainly in raw, unprocessed forms), by a reduced consume of meat and meat-derived products, and low to moderate intake of dairy products. Other distinctive characteristics are a moderate/high consume of fish and a high intake of unsaturated added lipids, especially in the form of olive oil [30].

However, attention on other healthy nutritional models, such as DASH Diet and Nordic Diet, is increasingly growing [53–56]. The characteristics of these diets are reported in Table 1. All these dietetic models imply a wide consume of fresh fruit and vegetables, whole cereals, pulses, fish, nuts and a limited intake of meat, dairy products and simple sugars [24, 57, 58] (https://sip.it/wp-content/uploads/2017/10/POSTER_PIRAMIDE.pdf). Holven et al. recently compared dietary intake and lipid values of Spanish and Norwegian children with FH following Mediterranean and Nordic Diet models, respectively. They concluded that nutrition advice should be more adapted to local intake patterns than on specific nutrient composition [17].

**Table 1:** Comparison of different healthy dietary patterns for prevention of cardiovascular disease

**Mediterranean diet**	**Nordic diet**	**Dietary Approaches to Stop Hypertension (DASH) diet**
Based on Greece and Southern Italy traditional diet. Refers to olive oil as principal dietary fat sources	Based on locally grown, seasonal food typical of the Nordic countries	Originally develop and used in the DASH trials to lower blood pressure
High in fibre intake and mono saturated fatty acids, white low in saturated fat	Rich in dietary fibre, ow in sugar and sodium	Rich in dietary fibre, potassium, calcium, low in saturated fat, restricted in sodium
• High intake of plant-based foods, such as fresh fruits, vegetables, legumes and nuts	• High intake of apple pears and berries, root, cabbages, cruciferous vegetables, potatoes, nuts and seeds	• Abundant amount of fruits and vegetables, pulses, nuts and seeds
• Daily intake of cereals (especially whole grain)	• High intake of whole grain oats and rye bread	• Regular consumption of whole grain
• Moderate consumption of fish (mainly blue fish)	• High intake of fish and seafood	• Fish intake
• Moderate consumption of dairy products (yogurt and cheese)	• Prefer low fat dairy products	• Increase consumption of fat free or low fat dairy products, reduced full-fat dairy products
• Low intake of red/processed meat; prefer chicken turkey. Sheep, goat	• Rarely red meats	• Prefer poultry to processed meat; limit high fat meat
• Olive oil as main source of culinary fat	• Use of vegetable fat (margarine, rapeseed oil)	• Use vegetable oils; limits tropical oils
• Low intake of sweets; wine allowed with meals, in moderation	• Avoid sugar-sweetened beverages or added sugar	Limits sweets and sugar sweetened foods and beverages

### Secondary hypercholesterolaemia

Hypercholesterolaemia in childhood and adolescence can be primitive, that is to say caused by a genetic alteration in cholesterol metabolic pathways, or secondary to other clinical conditions [59]. In case of secondary hypercholesterolaemia, the first therapeutic approach is the treatment of the underlying medical condition, such as hypothyroidism, liver disease, kidney disease, immunologic disease, and weight excess. In particular, overweight and obesity are epidemically increasing among children and adolescents. According to the Italian Consensus on obesity in childhood, approximately one every two children/adolescents with obesity has a dyslipidaemic pattern. The most frequent alterations of the lipid profile include elevated triglycerides levels and low HDL-C levels. Obesity related dyslipidaemia is secondary to the weight excess, therefore the first treatment is trying to reach ideal weight. According to this Consensus, paediatric patients with obesity that persistently have triglycerides higher than 500 mg/dl or LDL-C higher than 160 mg/dl, despite nutritional and lifestyle intervention, need a special paediatric lipidology evaluation [60]. Dietary approach for overweight and obesity in childhood and adolescence without a suspect of coexisting primitive hypercholesterolaemia are summarised in Table 2.

**Table 2:** Dietary approach for overweight and obesity in childhood and adolescence (adapted from Italian Consensus for Obesity in Childhood [60])

NUTRITIONAL MANAGEMENT OF CHILDREN/ADOLESCENTS WITH OVERWEIGHT AND/OR OBESITY
• Assessment of the child/adolescent’s and his/her family nutritional habits
• Investigate on meal composition, portions, weekly food frequency, cooking methods, food presentation
• Start with dietary avice
DIETARY ADVICE FOR CHILDREN/ADOLESCENTS WITH OVERWEIGHT AND/OR OBESITY
• Eat three meals and no more than two snacks per day
• Consume adequate breakfast
• Do not nibble between main meals
• Avoid junk food
• Increase daily amount of fruit, vegetables and fiber-rich cereals
• Aged adequate portion

### Nutritional advice for children and adolescents with FH

#### General intervention

When dealing with children and adolescents, nutritional counselling and treatment must always take into account each age different energy requirements and taste characteristics. In 2015 EAS Document [3], the nutritional treatment for children with hypercholesteroalemia consisted mainly in general indications, so as to respect each Country own tradition, which has a great influence on children nutrition. In Italy, the Italian Society for Pediatric Nutrition (Società Italiana di Nutrizione Pediatrica, SINUPE) provided precise indications for paediatric subjects with hypercholesterolaemia [10], promoting a population approach, so as to spread correct nutrition throughout the paediatric population; also, an individualised approach to identify and treat subjects at high cardiovascular risk was promoted. According to the Reference Intake of Nutrients and Energy for the Italian Population (Livelli di Assunzione di Riferimento per Nutrienti ed Energia, LARN), the nutritional treatment is based on age specific energy and macronutrients requirements [61]. In Italy a healthy-heart diet should be prescribed according with the Italian Society of Human Nutrition reference values (LARN). Daily energy intake must always be age related; protein intake 0,94–0,97 g/Kg per day; carbohydrate: 45–60% (sugar < 15%), fat: 20–35% (saturated fatty acids < 10%, polyunsaturated fatty acids 5–10%) of daily energy intake; fiber 8,4 g/1000 kcal [61]. In the recently published document by the Italian Agriculture Research Council (Consiglio per la RicErca in Agricoltura, CREA), the main nutritional indications for the Italian populations, including children and adolescents, have been reported. The CREA document [62] includes all the most recent nutritional issues, such as the consume of cereals other than wheat, the use of biological food, the environmental impact of nutrition and the multi-ethnical aspects of the current population. In our Country, Mediterranean diet and model represent a milestone, with partial contaminations from imported foods, such as cereals different from wheat, and exotic fruits [63].

#### Children and adolescents with FH

**Fig. 3:**
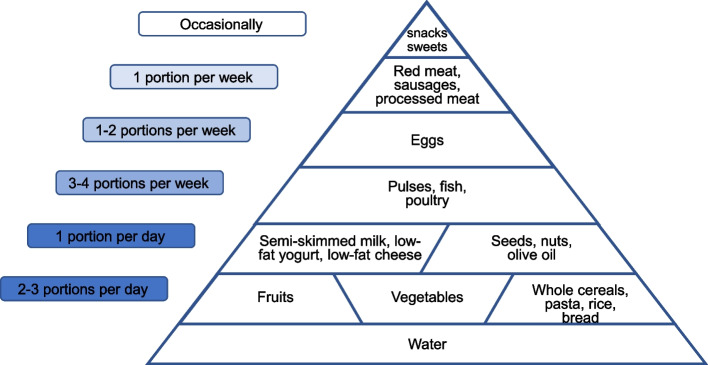
Main nutritional indications for paediatric subjects with FH, adapted from (https://sip.it/wp-content/uploads/2017/10/POSTER_PIRAMIDE.pdf)

Nutritional treatment for paediatric subjects with FH should grant an adequate energy intake, so as not to interfere with the growth pattern, meantime avoiding excessive restrictions [59]. Lipids represent a concentrated energy source, and they can be only partially limited: according to the main studies in this field, lipid daily intake should not be lower than 25–30% of total daily energy amount. Following the American Guidelines [2], nutritional intervention for FH in childhood is based on a two-step intervention: STEP ONE and STEP TWO diet, to be applied sequentially when approaching a paediatric patient with FH, as shown in Table 3. Protein intake should grant a solid energy basis for every child’s growth, but any excess should be avoided: in fact, we know that protein excess is linked to early development of overweight and obesity, which are further cardiovascular risk factors [60, 63, 64]. In the developmental age, the optimal protein intake should be between 12 and 14% of total daily energy amount, with a 1:1 ratio between animal and vegetal derived proteins. Carbohydrates are the main energy source, accounting ideally for 55 to 60% of total daily calories. An adequate amount of fibers should always be provided, and simple sugars should not exceed 10% of total daily calories, according to SINUPE [10]. Main nutritional indications are summarised in Fig. 3.

**Table 3:** Step One and Step Two Diet for paediatric patients with FH [1]

STEP ONE DIET
• Total fat ≤ 30% (no less than 20%) of total daily calories
• Saturated fats < 10% of total daily calories
• Polyunsaturated fats ≤ 10% of total daily calories
• Cholesterol ≤ 300 mg/day
STEP TWO DIET
• Total fat ≤ 30% (no less than 20%) of total daily calories
• Saturated fats < 7% of total daily calories
• Polyunsaturated fats ≤ 10% of total daily calories
• Cholesterol ≤ 200 mg/day

**Fig. 4:**
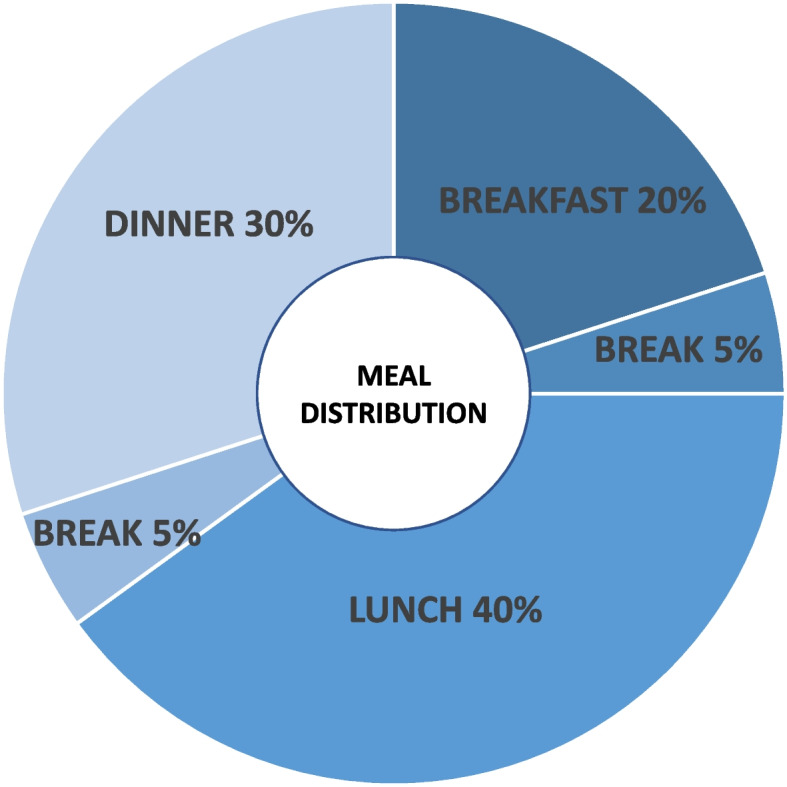
Meals distribution during the day, according to SINUPE guidelines [10]

An issue of utmost importance in paediatric patients is that meals should always be complete and all macronutrients (carbohydrates, lipids and proteins) should be present in every meal, so as to grant adequate intake of micronutrients as well. Calories distribution during the day is fundamental to support a harmonic growth, with no deficit or excess. Every day, food intake should be divided into four main meals plus one small break (breakfast, small break, lunch, afternoon break and dinner, as shown in Fig. 4). It is important to bear in mind that nutritional intervention for FH paediatric patients is not meant as a strict and mandatory diet, but it is based on all the nutritional counseling that all children and adolescents should follow for a healthy life [65]. That is why we highly recommend that children and adolescents with FH should not follow a special diet at school, whereas parents should compensate possible excessive school meals intake through the modulation of home-consumed and home-prepared meals. What is more, there are no “forbidden” foods: paediatric patients with FH can eat any kind of food, provided that junk food occasional assumption is balanced in the following meals with fruits, vegetables and healthy foods consume. In the clinical practice, this strategy, if reinforced and enhanced during periodical check-up and visits at the Lipid Clinic with both child/adolescent and parents, may help achieve a better compliance to the nutritional intervention proposed. FH children’s families are usually already sensitised to CVD prevention issues, as at least one parent is usually affected by hypercholesterolaemia. However, the active involvement of all family members in the daily management of a paediatric subject with FH makes this strategy a winning one. Therefore, the aim of nutritional intervention in paediatric patients with FH is to establish a correct, lipid controlled nutritional habit in the whole family, thus increasing the chances to last throughout adulthood.

### Frequent mistakes and possible risks

As we have previously highlighted, nutritional intervention in paediatric patients with FH is an issue of utmost importance and it should be intended as a combination of adequate lifestyle and nutritional habit. In this context, the involvement of all family members is a milestone in the daily management of paediatric patients with FH. Children and adolescents are growing-up subjects, therefore all meals should be complete in terms of macronutrients, so as to grant an adequate and healthy-heart nutrition. Traditionally, meals are a convivial moment that should be shared by all family members together, starting from breakfast, which is one of the most important meals of the day, and that should be prepared with care and consumed in a calm and relaxed context [66].

#### Food restriction

Adolescents should acquire healthy nutritional habits without stress, especially when dealing with food restrictions. In fact, a careless attitude and/or too restrictive dietary regimens may easily drive the child/adolescent into deviant thoughts about food and food choices, with a high risk of developing an eating disorder. Every time a healthcare professional gives advice on food restrictions and/or on food choices to limit lipid intake, the possible risk of leading a predisposed subject towards eating disorders must always be taken into account. This issue should be explained also to parents and to caregivers, so as not to stress the “limitation” aspect of nutritional intervention [20].

#### Calcium intake

When considering specific quantitative issues, the excessive limitation on milk, milk-derived and dairy products intake is one of the most frequent mistakes, often autonomously put into action by parents. Milk and dairy products are rich in cholesterol, but they also represent an important calcium source. Calcium could be defective as a consequence of an excessive restriction on this food category. Evaluation of calcium nutritional sources is an important step when analysing nutritional habits of paediatric patients with FH. In Table 4, the average calcium content of commonly consumed foods has been reported. We must remember that mineral (bottle) water is an important source of calcium as well, and mineral water can be used as an optimal and not expensive calcium source, in addition to other foods rich in this micronutrient. The average calcium content in tap water in Italy is 20.6–98 mg/dl (https://www.salute.gov.it/portale/temi/p2_6.jsp?lingua=italiano&id=4528&area=acque_potabili&menu=dieta). Calcium-rich mineral waters can reach calcium content of 400 mg/l. Considering that calcium Average Requirement (AR) for children aged seven to ten years is 900 mg per day [61], we propose an example of calcium-rich foods distribution in the main meals and breaks, so as to reach AR (see Table 5).

**Table 4:** Calcium content in foods. Data adapted from CREA [62]

FOOD	CALCIUM (mg/100 g)
Cow’s milk	1323
Parmesan cheese	1165
«Crescenza» cheese	557
«mozarella» cheese	350
Rocket salad	309
«Ricotta» cheese	295
Almonds	240
Dried figs	186
Baked anchovies	165
Chicory	150
Beans	135
Goat yogurt	124
Semi skimmed cow milk yogurt	120
Radish	115
Artichoke	86

**Table 5:** Example of a possible distribution of calcium-rich foods in the daily meal organization of a 8 years old child (energy average requirement for a male 8 year old child with medium physical activity is 1870 kcal/day, calcium average requirement for a 8 year old child is 900 mg/day, according to LARN [61])

Meal	Calcium rich food	Calcium (mg)
Breakfast	One greek yogurt (125 mg)	188
Break	20 almonds	72
Lunch	Rocket salad 80 g	247
	200 ml of calcium-rich water	80
Break	3 dried figs	56
Dinner	Beans (100 g)	135
	Parmesan cheese on first course (10 g)	116

#### Polyunsaturated fatty acids

Polyunsaturated fatty acids (PUFAs) are a group of fatty acids that contain more than one double bond in their molecular structure. The most important PUFA groups are omega-3 (n-3) and omega-6 (n-6), depending on the placement of the first double bond, which is either at the third or the sixth carbon of the methyl end. Long chain polyunsaturated fatty acids (LCPUFAs) are fatty acids, other than linoleic acid, gamma linolenic acid and alpha linolenic acid, which contain at least eighteen carbon atoms and at least two double bonds [9]. In cardiovascular prevention field, omega-3 and omega-6 PUFA are the most studied fatty acids, as they can modulate lipid plasma values and inflammatory markers implied in the atherosclerotic process. LCPUFAs adequate intake should be granted in FH paediatric patients’ nutrition. LCPUFAs are contained in fish, especially blue fish, but also in seeds and nuts. In the clinical practice, we must consider that fish can be an expensive food, not affordable by all families, therefore alternative sources of LCPUFAs should be proposed when making a nutritional counselling for all family members.

Content of LCPUFAs in different kinds of fish and in bred and wild fish are reported in Tables 6 and 7 [67].

**Table 6:** LCPUFA content in fish species consumed in Italy, adapted from [67]

Fish species	Anchovies	Sardine	Hake	Trout	Eel	Pangsius
Total pufa (g/100 g)	45.96	36.32	40.70	31.24	17.40	15.55
Sum of (n-3)	42.66	33.50	35.90	24.62	9.72	4.43
Sum of (n-6)	3.30	2.82	4.80	6.61	7.66	11.11
(n-3) (n-6)	12.9	11.9	7.3	3.72	1.26	0.40

**Table 7:** Lipid and long chain unsaturated fatty acids content (total lipid expressed as g/100 g of edible part, fatty acids expressed as percentage of total fatty acids) in bred and wild species of fish consumed in Italy, adapted from [67]

Fish species	Bread sea bass	Wild sea bass	Bred daurade	Wild daurade	Bred salmon	Wild salamon
Total lipid content (g)	9.36	2.15	11.13	7.37	12.3	6.3
Total PUFA (g/100 g)	30.89	31.50	30.70	16.48	300.4	26.2
C 22: 6n-3	10.79	12.06	10.93	3.31	8.4	12.5
Sum of (n-3)	22.70	23.66	24.07	12.06	21.2	24.4
Sum of (n-6)	8.19	7.84	6.64	4.42	9.2	1.8
(n-3) (n-6)	2.77	3.02	3.63	2.73	2.3	13.6

Nuts are a good source of LCPUFAs’ precursors and can be considered for snacks or breaks, provided that the adequate portion is defined, as reported in Table 8 [62].

**Table 8:** Average portion of nuts (30 g) for primary school children (adapted from CREA [62])

Type of nuts	Number of pieces per portion
Nuts	8
Almond	25
Hazelnut	28
Pistachios	50

#### Fibres

The consume of whole and/or organic foods has widely spread in the past decades, with low risk of excesses, as we seldom witness excessive fibre consume in FH patients’ families. On the contrary, daily consume of whole pasta, cereals and bakery products is often not easy to be accepted.

Nutritional evaluation of paediatric patients with FH must take into account adequate energy and macronutrients intake and macronutrients and micronutrients correct balance, avoiding excess and strict limitations. The patient’s age, taste and preferences, family’s habits and traditions and socio-economic and cultural background are fundamental milestones that have to be considered when nutritionally approaching FH patients. All these aspects must be tightly connected and reinforced through regular and frequent nutritional counselling. Nutritional habits should be built with a “step by step” approach, modifying little aspects of nutrition at each visit and promoting active participation of the child/adolescent in his or her own nutritional choices [20, 65].

## Conclusive considerations

Nutritional intervention is the first-step intervention in the treatment of paediatric patients with dyslipidaemia, also in severe forms such as FH. In the last years, research in nutritional field has highlighted the importance of dietary patterns instead of single nutrients for the impact on lipid profile and on CVD prevention. Starting from main consensus documents indications, we have analysed macronutrients intake, evaluating each macronutrient peculiar characteristic and trying to insert them into a balanced dietary pattern, according to main national and international documents. Adequate energy intake, consume of complete meals, products quality but also environmental sustainability and feasibility in the cultural and traditional family context are all pieces of the complex puzzle that Pediatric Lipidologists have to compose.

In Italy, the Mediterranean Diet is the most widely accepted and feasible one, as it provides a large amount of fruits and vegetables, pulses, whole cereals, extra virgin olive oil, and a reduced and controlled intake of meat, preferring white meat and poultry, and eggs, fish, milk and dairy products. Finally, we have analysed some specific quantitative aspects of the nutritional recommendations for patients with FH and the most common risks and/or mistakes when dealing with FH paediatric nutrition, including psychological aspects, and the importance of a tailored approach, according to each family socio-economic and cultural background.

In conclusion, nutritional intervention for children and adolescents with FH cannot be reduced to a standardised and strict dietetic scheme, but it should be modulated, tailored, personalised and periodically renewed through each patient’s counseling. Taste, nutritional habits and family’s traditions and cultural background should always be taken in consideration. The active involvement of the child/adolescent and of the whole family should ensure good adherence and the establishment of correct healthy-heart habits that will possibly last throughout adulthood.

## Data Availability

Not applicable.
